# Mpox Virus: Its Molecular Evolution and Potential Impact on Viral Epidemiology

**DOI:** 10.3390/v15040995

**Published:** 2023-04-18

**Authors:** Xi Yu, Huicheng Shi, Gong Cheng

**Affiliations:** 1 Tsinghua-Peking Center for Life Sciences, School of Medicine, Tsinghua University, Beijing 100084, China; 2 Institute of Infectious Diseases, Shenzhen Bay Laboratory, Shenzhen 518000, China; 3 School of Life Sciences, Tsinghua University, Beijing 100084, China; 4 Institute of Pathogenic Organisms, Shenzhen Center for Disease Control and Prevention, Shenzhen 518055, China

**Keywords:** mpox virus, molecular evolution, epidemiology

## Abstract

Mpox (previously known as monkeypox) is an infectious viral illness caused by the mpox virus (MPXV), an orthopoxvirus that belongs to the family *Poxviridae*. The symptoms of mpox in humans are similar to those of smallpox, although the mortality rate is lower. In recent years, the concern over a potential global pandemic has increased due to reports of mpox spreading across Africa and other parts of the world. Prior to this discovery, mpox was a rare zoonotic disease restricted to endemic regions of Western and Central Africa. The sudden emergence of MPXV cases in multiple regions has raised concerns about its natural evolution. This review aims to provide an overview of previously available information about MPXV, including its genome, morphology, hosts and reservoirs, and virus–host interaction and immunology, as well as to perform phylogenetic analysis on available MPXV genomes, with an emphasis on the evolution of the genome in humans as new cases emerge.

## 1. Introduction

Mpox (previously known as monkeypox) is a zoonotic viral illness caused by the mpox virus (MPXV), an orthopoxvirus within the *Poxviridae* family. The probable origin of the term ‘monkeypox’ likely derives from the fact that MPXV was first identified in 1958 in Singapore-shipped study monkeys [1]. The variola virus (VARV), the causative agent of the fatal smallpox disease, is also a member of the genus Orthopoxvirus. The symptoms of mpox in humans resemble those of smallpox, but with a reduced death rate [2,3]. In the 1970s, sporadic cases of MPXV in humans were detected in a number of African countries; however, over the past two decades, the virus has spread more widely across the continent.

Recently, mpox has made headlines around the world and raised fears of a new global pandemic. Over 110 countries on 6 continents have reported cases, and the Centers for Disease Control and Prevention (CDC) has issued a level two alert [4]. Prior to this discovery, mpox virus (MPXV) was a rare zoonotic disease restricted to endemic regions of Western and Central Africa. Two distinct clades of MPXV can be distinguished phylogenetically: Central African (also known as the Congo Basin) and West African. All known non-African cases, including those currently circulating, have been attributed to the West African clade [5,6]. Currently, it is being investigated whether genetic alterations in the MPXV genome are to blame for the current outbreak [7].

In light of the recent increase in mpox cases, we provide a narrative review of previously available information about MPXV, as well as performing phylogenetic analysis on available MPXV genomes, with an emphasis on its evolution in humans as new cases emerge.

## 2. Mpox Virus

### 2.1. Introduction to the Family Poxviridae

Poxviruses are large DNA viruses that infect a diverse array of hosts. The *Poxviridae* family is separated into *Chordopoxvirinae*, which infect vertebrates, and *Entomopoxvirinae*, which infect insects [8,9]. *Orthopoxviruses* from the *Chordopoxvirinae* genus include the human pathogens variola virus (VARV), which causes smallpox, and the mpox virus (MPXV) as well as the vaccinia virus (VACV). All *Orthopoxviridae* induce humoral responses that are cross-reactive in addition to cellular immune responses.

**Figure 1:**
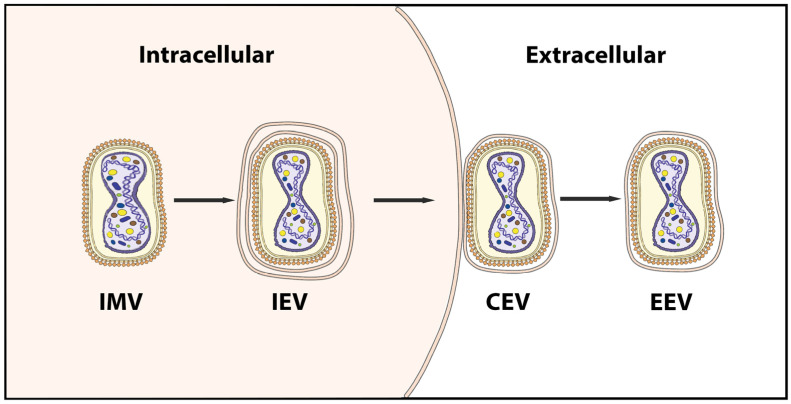
Schematic of the four infectious virion forms of poxviruses produced during the virus life cycle. IMV, intracellular mature virus; IEV, intracellular enveloped virus; CEV, cell-associated enveloped virus; EEV, extracellular enveloped virus.

Orthopoxviruses are enveloped, double-stranded DNA viruses with a genome encoding ~200 genes and a size of 180–220 kilobase pairs (kb). The virions are around 250 nm × 220 nm in size. A low-pH-dependent macropinocytotic uptake releases the viral core into the cytoplasm, enabling viral entrance into the cell [10]. Initiation of early gene expression and virus uncoating occurs in the viral core [11]. This results in DNA replication followed by gene expression at the intermediate and late stages. The assembly of DNA molecules, viral enzymes, and structural proteins produces mature viral particles [12]. The two infectious mature forms of poxviruses are the extracellular enveloped virus (EEV) and the intracellular mature virus (IMV) (Figure 1). It is believed that EEV facilitates the spread of the virus within an infected organism, whereas IMV facilitates transmission between hosts [12,13,14].

### 2.2. Genome and Morphology of MPXV

The MPXV genome is about 197,000 kb in size and contains more than 190 open reading frames (ORFs) [15,16]. The highly conserved central coding region of the genome is flanked by diverse ends that contain inverted terminal repeats. At least 90 open reading frames (ORFs) are necessary for the replication and morphogenesis of poxvirus. Many so-called non-essential ORFs play a role in the differences in poxvirus host tropism, immunomodulation, and pathogenesis, and many ORFs have yet to be functionally characterized [17]. The average size of MPXV virions is 280 nm × 220 nm, and they are shaped like barrels or ovals [18]. Poxvirus mature particles have a distinctive dumbbell-shaped nucleoprotein core that contains a large double-stranded linear DNA genome [18]. In addition to virus-encoded DNA-dependent RNA polymerases and related transcriptional enzymes, MPXV virions contain over 30 structural and membrane viral proteins [19,20].

The central portion of the MPXV genome contains genes known to be essential for orthopoxviruses [15,21]. However, in contrast to other orthopoxvirus genomes, a minority of ORFs are lost or truncated in the MPXV genome [15,22,23]. Several ORFs encoding genes involved in immune evasion have been identified as being disrupted in the West African clade; these mutations may account for the lower virulence of this clade compared to the Central African clade [22,23,24].

The virus exists in two distinct infectious forms, namely the intracellular mature virus (IMV) and the extracellular enveloped virus (EEV), whose surface glycoproteins and cell-infecting mechanisms are distinct [25]. EEV, which is believed to be responsible for early dispersal, and IMV released during cell lysis are both capable of mediating infection [26,27]. The fundamental structural distinction between IMV and EEV is that IMV lacks the additional outermost membrane layer. The amounts of viral proteins incorporated into the two types of virions also differed [20,26].

MPXV replication is a complicated process, although it is widely believed to be the same as that of other orthopoxviruses [25]. MPXV entry receptors have not been definitively discovered; however, it has been hypothesized that viral entry is dependent on viral strain and host cell type and involves numerous surface receptors, such as heparan sulfate or chondroitin sulfate [28,29,30]. The surface proteins H3, D8, and A27 have been linked to viral binding in VACV [28,29,30]. Eleven conserved proteins create a complex known as the entrance fusion complex, which allows VACV to gain access into the cell after binding [31].

### 2.3. Hosts and Reservoirs

MPXV has been discovered in numerous species, but it remains unclear as to which of these species acts as the primary animal reservoir. Tissue and host tropism have a significant impact on the virus’ dissemination and propagation inside an infected host and between hosts [25]. Numerous mammalian species have been spontaneously infected with MPXV, despite the fact that its reservoir host has not been discovered [32,33,34,35,36,37]. Thus, it is assumed that MPXV has a broad host range. A study discovered large quantities of viral DNA and live virions in a variety of organs in animals that died after being exposed to Congo Basin MPXV, which may indicate a broad tissue tropism [38]. There is no clear reservoir or natural host for MPXV, although it has been discovered that rodents and non-human primates are possible natural reservoirs and incidental hosts [39,40,41,42].

## 3. Virus–Host Interaction and Immunology

### 3.1. Innate Immune Responses to MPXV

Typically, innate immune cells serve as the initial line of defense following an active viral infection; nevertheless, these cells are also targets for certain viruses. Numerous in vitro and in vivo studies have established that poxviruses target monocytes first [43,44,45,46,47]. Similar to monocytes, natural killer cells are an essential component of innate immunity [48]. In rhesus macaques infected with MPXV, natural killer cell counts increase dramatically in peripheral blood and the lymph nodes [49]. Prior to this fast expansion, MPXV infection greatly inhibited the migratory capacity of the major natural killer cell subsets, which severely hindered their recruitment into lymphoid and/or inflammatory tissues [49].

Numerous innate immune cells, including macrophages, neutrophils, natural killer cells, conventional dendritic cells, plasmacytoid dendritic cells, and innate lymphoid cells, play unknown functions in MPXV-infected patients. The characterization and profiling of these immune cells during MPXV infection will be crucial for elucidating their activities and developing prognostic biomarkers. It has been shown that human IFN-β inhibits MPXV replication and spread [50]. However, MPXV did not strongly activate TNF-regulated and NF-κB-regulated genes, particularly in infected animals [45]. Numerous cytokines are raised in reported cases of human MPXV infection [51]. By suppressing inflammatory and antiviral immune responses, VACV is able to evade immunological responses, and MPXV may use a similar strategy to trick the host immunity [52,53,54,55].

### 3.2. Adaptive Immune Responses to MPXV

With the successful worldwide immunization effort that eradicated smallpox with a live VACV vaccine, the relevance of B cells and immunoglobulins against poxviruses was proven [56,57]. VACV-specific B cell responses were instrumental in shielding rhesus macaques from a fatal MPXV infection [58]. Importantly, epidemiological studies have shown that the VACV vaccine protects against additional poxviruses, including MPXV [59]. In several cases, the VACV-specific memory B cells and antibody levels generated by immunization lasted longer than 50 years [60,61]. However, only 50% of vaccinated patients had neutralizing antibody titers sufficient for conferring protective immunity against smallpox after 20 years [44,62]. It is probable that cross-protective immunity against mpox will also diminish over time.

CD4+ T cells, specifically T follicular helper cells, have a role in promoting the recall and differentiation of memory B cells into antibody-producing cells [63]. Following vaccination with VACV, memory CD4+ T cells were found to remain for at least 50 years, with an estimated half-life of 8–15 years [61]. It has been demonstrated that the amount of CD4+ T cells is crucial for eliciting a protective antibody response against deadly MPXV infection in vaccinated rhesus macaques [58]. T cells can have direct antiviral roles in addition to assisting in the generation of antibodies. Given that orthopoxviruses, including MPXV, infect and spread in macrophages, cytolytic T cells can play a crucial role in eradicating infected macrophages to prevent viral propagation [43,44,45,46,47]. CD8+ T cells have been shown to destroy virus-infected monocytes and inhibit virus propagation in a mouse model of VACV infection [64]. However, the connection between CD4+ and CD8+ T cell responses and the severity of MPXV infection remains ambiguous in human studies.

### 3.3. Immune Evasion

Poxviruses adopt a variety of strategies to prevent identification and removal by the immune systems of their hosts. In primary fibroblasts, MPXV infection does not increase interferon-stimulated gene expression and lowers TNF-α, IL-1, IL-6, and CCL5 activation [47]. Multiple MPXV-encoded proteins assist immune evasion (Table 1). For instance, viral protein B16 inhibits antiviral signaling induced by type I interferon [65]. A homolog of D7L has been discovered to block the proinflammatory cytokine interleukin 18 (IL-18) which is required for the regulation of mpox viraemia in mice [66]. ZAP targets CpG dinucleotides in RNAs and exerts a selective pressure on CpGs in viral genomes [67,68,69]. However, neither the MPXV genome nor mRNAs are inhibited by ZAP. The C16 protein of VACV has been shown to bind ZAP and decrease its antiviral activity, and its homolog in MPXV may play a similar role [70]. Another example of an MPXV immunomodulator is complement control protein (CCP) which inhibits the initiation of the complement activation pathway [71]. As it lacks the CCP gene, the West African (WA) clade has a lower case fatality rate than the Congo Basin (CB) clade [72]. The removal of CCP from the CB MPXV strain reduced the incidence and mortality of prairie dog illness [71]. However, additional characteristics not yet identified contribute to the pathogenicity difference between the WA and CB clades. As many additional MPXV ORFs need to be functionally characterized, our knowledge of MPXV immunomodulators is still insufficient.

## 4. Transmission and Epidemiology

### 4.1. Transmission

Contrary to its name, MPXV is not originated from monkeys. Humans and monkeys are merely incidental hosts and the reservoirs are thought to predominantly include rodents [73]. The transmission of MPXV from animals to humans is well documented and often happens through contact with body fluids or a bite [74]. The systemic disease was more likely to develop in patients with an invasive bite from an infected animal than in those with noninvasive exposures. Large respiratory droplets, extended face-to-face contact, close touch with infectious skin lesions, or bodily fluids are typically required for human-to-human transmission to take place. Risk factors for viral transmission also include contaminated objects and surfaces, such as sharing a home, sleeping in the same bed, or eating or drinking from the same dishes as an infected person [75]. The virus may be transmitted from the mother to the fetus through the placenta [75]. Although there are issues over the viability of aerosol delivery of MPXV, there is no evidence to support this at this time. Despite the fact that cases of mpox associated with sexual contact are more likely to be the result of direct contact with skin lesions than sexual transmission, the latter theory was proposed after seminal fluid samples from some cases tested positive for MPXV [76]. Nonetheless, the clinical significance of this remains unknown.

### 4.2. Epidemiology

MPXV was initially isolated in 1958 in Copenhagen, Denmark, following two outbreaks of a nonfatal rash disease in Singaporean cynomolgus macaques held in captivity [77]. In the decade that followed, similar epidemics were observed in European and American primate colonies [1]. MPXV was not identified as a human disease until the first human case was discovered in 1970. During the late phase of the global smallpox eradication campaign, rigorous surveillance for smallpox-like diseases in West and Central Africa found the first case of a child in the Democratic Republic of the Congo (DRC) [78,79]. Six further cases were later discovered in Liberia, Nigeria, and Sierra Leone [80].

Prior to the epidemic in the USA in 2003 when infected rodents were unintentionally transported there, mpox was not known to exist outside of Africa [18]. A total of 34 laboratory-confirmed human mpox cases were found at that time, totaling 71 cases across 6 states [81]. The disease appears to have a relatively low rate of person-to-person transmission during that outbreak [82]. Even before the current outbreaks, concern regarding the impact of mpox on public health was mounting due to the disease’s geographic spread and subsequent comeback in regions where cases had not been reported in decades [72].

From 2018 to 2021, four individuals in the UK were diagnosed with travel-associated MPX which was then transmitted to three additional patients [83]. The first household cluster outside of Africa was noted at this time. According to the UK, another instance of mpox outside of Africa was discovered on 7 May 2022 in a traveler coming back from Nigeria. Numerous instances have now been discovered, many of which lack epidemiological connections to the imported case from Nigeria or to the family cluster. However, it has been noted that many of these new cases involved men who have sex with men (MSM) who frequently displayed symptoms of a rash resembling a vesicular disease, lymphadenopathy, and fever [84]. More than 85,000 people have been affected by the disease so far, with cases recorded in 110 countries mostly not typically endemic for MPXV, and in some cases with no known travel connection [4,85].

The recent cases demonstrated a quick human-to-human transmission, prompting fears for a rapid community expansion. Since the majority of the patients did not visit the endemic regions of Africa, it is plausible that community transmission has gone undetected in the past. Additionally, the fact that it spread to other nations at the same time shows that there were numerous sites of introduction and transmission. These outbreak-related topics require more in-depth research to be able to be answered.

The reasons for the resurgence of mpox cases have been hotly debated with declining immunity being the most popular theory [86,87,88]. Deforestation might potentially contribute to the problem or perhaps act as a potentiator. Mpox was unknown during the period when smallpox was common. This might have happened because the two diseases present similarly and the focus was on smallpox or because the absence of laboratory evidence of the etiologic agent led to the assumption that smallpox was the cause [89]. Another potential element influencing the recurrence of the disease is the genetic evolution of the mpox virus which will be discussed in detail in the next section.

## 5. Phylogeny and Evolution

The evolution of zoonotic infections to become more transmissible or virulent in humans is a key cause for concern. Particularly, regarding OPXVs such as MPXV, there is concern about the risk that they could evolve into infections capable of causing another smallpox-like pandemic. Reviewing the evolution mechanisms of poxviruses, some of which are highly unusual among viruses, can provide insight into the MPXV’s evolutionary potential.

### 5.1. Phylogeny

In contrast to smallpox which has only ever been associated with humans, MPXV can infect both animals and humans [32]. The virus can be separated into the Central African clade and the West African clade based on genetic and geographic variation [90]. Based on the higher mortality rates that have been observed, the Central African clade appears to be more virulent [91].

**Figure 2:**
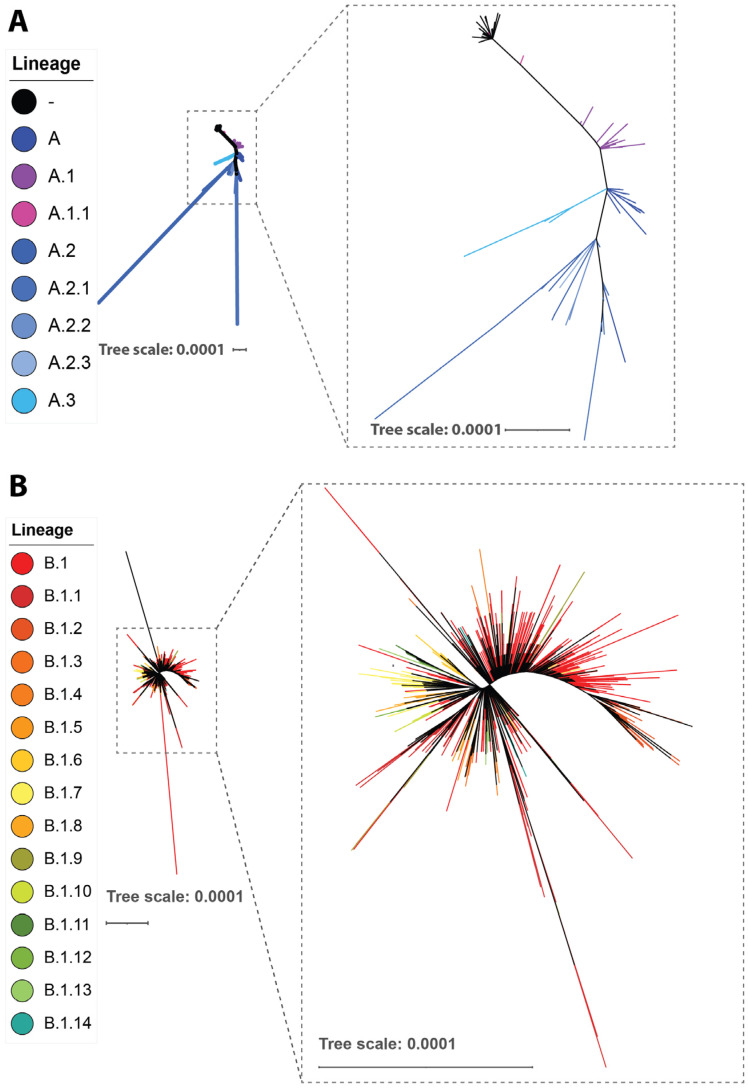
Phylogenetic analysis of MPXV Clade 3 lineage A (**A**) and lineage B (**B**). Genomic sequences were downloaded from GISAID (https://gisaid.org/) (accessed on 7 December 2022) with their metadata, including the date and location that the sequences were collected. The sequences were aligned and translated by nextalign using an annotated pre-outbreak sequence (NCBI Reference Sequence: NC_063383.1, GISAID Accession ID EPI_ISL_13056282) as the reference sequence. The clade that each sequence belongs to was assigned using nucleotide substitutions defined by the Nextstrain team at https://github.com/nextstrain/monkeypox/blob/master/config/clades.tsv (accessed on 7 December 2022) [92]. A custom script was used to identify nucleotide and amino acid substitutions in each sequence compared to the reference so the date that a substitution first appeared and the frequency of the substitution in the years before the outbreak and months after the outbreak can be obtained. The aligned genomes were trimmed with trimAl for phylogenetic analysis using IQ-TREE [93,94]. The maximum likelihood trees of both Lineage A and B were inferred with the automatic ModelFinder and ultrafast bootstrap [95,96]. The resulting trees were visualized with the Interactive Tree Of Life (iTOL) [97,98].

We examined MPXV genomes from GISAID and NCBI that correspond to the various MPXV outbreaks and contain virus sequences from animal reservoirs and earlier human outbreaks. These MPXV genomes diverged into three major clades identified for MPXV: the MPXV Clade 1 from the 1970 to 2017 outbreaks in Central Africa; the MPXV Clade 2 from the 1970 to 2017 outbreaks in different nations; and the MPXV Clade 3 from the recent MPXV epidemic outbreaks (2017–2022). The latter clade consists of the newly classified lineages A.1, A.1.1, A.2, and B.1, with lineage B.1 comprising all the MPXV genomes from the 2022 outbreak (Figure 2). Thus, we focused our further analysis of single-nucleotide polymorphisms (SNPs) in Clade 3.

Even though the Central African clade is more frequent, outbreaks in the United States and Nigeria account for the majority of West African clade cases. This was also found in instances involving travel to Israel, Singapore, and the United Kingdom [99,100,101]. All cases reported in the United Kingdom during the current outbreak have been attributed to the MPXV West African clade, and all MPXV genomes from the 2022 mpox outbreak belonged to this clade [85].

The 2022 outbreak cluster, lineage B.1, is a divergent branch that descends from a lineage A.1 linked to MPXV exports in 2018 and 2019 from Nigeria to the United Kingdom, Israel, and Singapore, with genetic ties to a sizable outbreak that occurred in Nigeria in 2017–2018 [90,91,102]. Given these findings and the historical epidemiology of the MPXV, it is likely that the MPXV imported from an endemic country caused the outbreak that broke out in 2022. The MPXV detected in 2022 may also represent the ongoing spread and evolution of the virus that caused the outbreak in 2017–2018 in Nigeria.

### 5.2. SNPs

As its DNA genome is replicated by a viral DNA polymerase with 3′−5′ exonuclease proofreading activity, the mutation rate of poxvirus is lower than that of RNA viruses [103]. The estimated substitution rate of poxviruses from the molecular clock study is between 2 × 10^−6^ and 1 × 10^−5^ nucleotide substitutions per site per year, which could result in up to 2 nucleotide alterations in the genome every year [104,105]. Comparatively, the substitution rates for RNA viruses range from 10^−2^ to 10^−5^ nucleotide changes per site per year [106].

The genomes of the initial MPXV isolate from West Africa in 1971 and of strains from the outbreak in 2022 change by less than 0.06%. Analysis of the nucleotide makeup of the MPXV genome indicated that its AT content is approximately twice as high as its GC content [107]. It is well recognized that mammalian DNA and RNA binding or editing enzymes impose selective pressures on viral genomes, frequently causing a bias in genomic nucleotide utilization. APOBECs, for instance, can accelerate viral mutation rates, resulting in a drop in C content and an increase in T content as a result of cytosine deamination [108,109]. Early investigations demonstrated that APOBEC3 family members have no effect on the short-term replication of VACV [110], but assessments of MPXV genomes from previous years and the ongoing 2022 outbreak revealed that 90% of new nucleotide alterations were indicative of APOBEC3 editing [107].

In order to quickly gain the first insights on phylogenetic placement and evolutionary tendencies of the MPXV that causes the recent outbreak, we concentrated our research on the first outbreak related MPXV genome sequence which was publicly disclosed in 2018 as well as on subsequent sequences released in NCBI and GISAID before September 2022. The sequences were aligned and translated by nextalign using the annotated pre-outbreak sequence (NCBI Reference Sequence: NC_063383.1, GISAID Accession ID EPI_ISL_13056282) as the reference sequence. The clade that each sequence belongs to was assigned using nucleotide substitutions defined by the Nextstrain team [92]. A custom script was used to identify nucleotide and amino acid substitutions in each sequence compared to the reference, so the date that a substitution first appeared and the frequency of the substitution in the years before the outbreak and months after the outbreak can be obtained (Supplementary Table S1).

**Figure 3:**
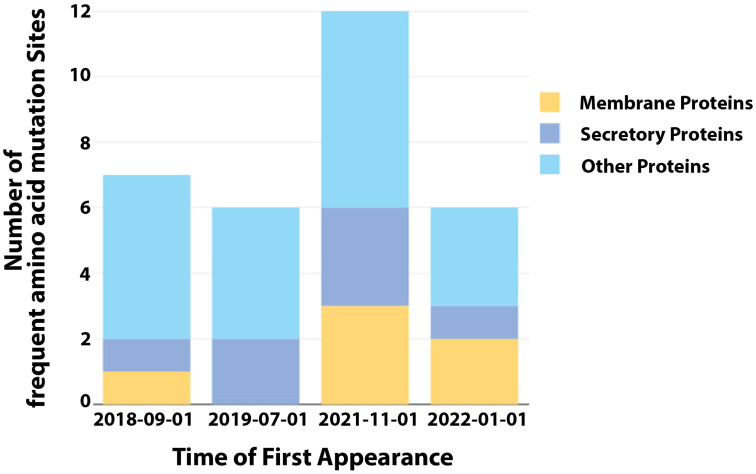
The incidence of frequent amino acid mutations. The frequency of the appearance of SNPs in different months in 2022 was analyzed and 31 frequent non-synonymous mutations were found. The frequent amino acid mutations were clustered into four groups based on their dates of first appearance, which were determined through evolutionary analysis. The mutations located in different viral proteins are indicated by colors. The data of record numbers are sourced from Table 1.

Notably, the 2022 MPXV diverges from the comparable 2018 virus much faster than expected based on prior estimates of the substitution rate for Orthopoxviruses (1–2 substitutions per genome per year) [111]. This divergent branch could indicate rapid evolution. Among these SNPs, we analyzed their frequency of appearance in different months in 2022 and found 31 frequent non-synonymous mutations (Table 2). The frequent amino acid mutations were clustered into four groups based on their dates of first appearance which were determined through evolutionary analysis. Notably, the incidence of amino acid mutations that are conserved in the MPXV genomes over time has increased significantly since November 2021 (Figure 3). The occurrence of multiple genetic changes within a brief period, which is not frequently observed in orthopoxviruses related to MPXV, implies that the virus has rapidly adapted to its host and gained a fitness advantage that helps to maintain human-to-human transmission [104]. This rapid sequence of alterations may have allowed the virus to quickly adapt to new hosts or environmental conditions, resulting in a higher likelihood of transmission and establishment in the human population. Such an ability to quickly adapt and evolve may be critical for the virus to persist and spread in a dynamic and changing environment.

### 5.3. Recombination

During cell infection, poxviruses undergo high-frequency recombination [112,113]. There has been naturally occurring intra-species recombination between VARV and VACV strains [114,115]. Recombination is thought to be one of the main forces driving poxvirus evolution [112]. The 3′-to-5′ exonuclease activity of viral DNA polymerases plays a crucial role in inducing genetic recombination during vaccinia infection [116]. This genetic recombination allows the vaccinia virus to adjust and resist the antiviral response of the host cells that is triggered by Protein Kinase R (PKR). Overwhelming evidence suggests that tandem gene duplications are the product of recombination [117,118]. However, spontaneous recombination events involving MPXVs are seldom described. A recent study reports the first natural recombination of the MPXV genome using SNP-dependent and SNP-independent analysis tools, namely linkage disequilibrium (LD) and tandem repeat (TR) analysis [119]. The authors speculate that the progeny MPXV recombinants emerged from a single origin, gained mutations, evolved into different lineages, and then underwent homologous recombination through multiple possible mechanisms. The resulting recombinants had mosaic patterns of TRs or mutations and no defective MPXV virus was detected arising from a single infection.

A study used homologous recombination to replace the D14L gene in the MPXV-Z genome with an EGFP-GPT cassette to assess the involvement of the monkeypox inhibitor of complement enzymes (MOPICE) in MPXV pathogenesis [120] and a recombinant MPXV producing green fluorescent protein was generated to analyze MPXV infection in a monkey model [121]. The possibility of constructing recombinant MPXVs in the laboratory increases the prospect that recombination between co-infecting MPXVs and naturally occurring OPVs may occur or has occurred in nature.

### 5.4. Gene Loss and Amplification

OPV evolution is additionally driven by variance in its genome content. The vast, adaptable genome of poxvirus enables substantial structural alterations that lead to gene loss or gene gain and alter viral behaviors more rapidly. OPVs adapt to their host by discarding and acquiring genes [122]. Researchers used the vaccinia poxvirus as a model and exposed it to serial propagation in human cells where the anti-host factor K3L is not effective against the anti-viral Protein Kinase R [123]. The viruses rapidly improved their fitness by repeatedly amplifying the K3L gene, resulting in up to 7–10% increases in genome size. These gene expansions were essential to counteract human PKR and allowed for an adaptive amino acid substitution in K3L that defeated PKR. The study also found that subsequent reductions in gene amplifications offset the costs associated with a larger genome size while retaining adaptive substitutions. This discovery explains how poxviruses can quickly adapt and overcome various host defenses despite having low mutation rates.

The genome-sequence length and gene content correlate positively with a broad host range but negatively with the pathogenicity [124]. The WA clade of MPXV has bigger genomes and more content than the CB clade, which may contribute to the WA clade’s lower pathogenicity [125]. This is supported by research conducted on ground squirrels which indicated that animals infected with Congo Basin MPXV had more severe symptoms and died sooner than animals infected with West African MPXV [126,127]. Strong evidence suggests that the primary mechanism for gene loss is the introduction of early stop mutations which lead to fragmentation, truncation, and total deletion of the ORF [128]. In a recent study, the genomes of five MPXV viruses from the 2022 outbreak in multiple countries were characterized, revealing gene duplications of up to 18,000 bp from both the left-to-right and right-to-left ITR regions [129]. This duplication led to gene deletions of up to 17,000 bp in the insertion region, suggesting that gene duplication and loss may be potential mechanisms of adaptation to the human host during the current MPXV outbreak. These findings highlight the importance of monitoring the genome ends, in addition to tracking non-synonymous mutations in future surveillance efforts.

## 6. Discussion

In the midst of the COVID-19 pandemic, the sudden emergence and escalating number of MPXV cases in non-endemic nations have sparked international concern [85]. With regard to the unusually enhanced human-to-human transmission among patients who have no prior travel history to endemic areas, the sudden emergence of MPXV cases in several regions has raised many concerns about the natural evolution of this current multi-country outbreak [130]. This suggests the existence of undetected transmission chains with multiple sources of introduction.

Multiple ideas have been proposed to explain the sudden increase in cases in Africa and other nonendemic regions. One theory argues that the end of mass smallpox vaccination in the 1980s, which conferred up to 85 percent cross-immunity against MPXV, enhanced human sensitivity to the virus [131,132]. In turn, this has imposed selective pressure on MPXV, encouraging the evolution of immune evasion mechanisms and resulting in an increase in the virus’ transmissibility. The acquisition of non-synonymous mutations associated with coding areas for expected host recognition elements could be a source of fitness adaptation for the virus, according to a second theory [107]. The occurrence of specific mutations in lineage B.1 genomes relative to those of related viruses in 2018–2019 cases has piqued the interest of researchers due to its segregation into this divergent phylogenetic branch and signaling accelerated micro-evolutionary events that may lead to increased human-to-human transmission [107].

The appearance of the lineage B.1 MPXV, which is responsible for the current outbreak on the European continent, was projected to have occurred as early as March 2022. This finding may indicate that B.1 developed and propagated across Europe, spawning the first cases of MPXV which then spread to other continents including Oceania and the Americas. Moreover, our analyses indicate that the MPXV has been adapting more rapidly over the past two years, suggesting accelerated adaptation to its host. The COVID-19 pandemic and multiple previous studies on viruses have demonstrated the importance of turning genetic results into useful techniques for tracking transmission patterns and predicting the advent of rapidly evolving diseases, such as the 2022-MPXV [133,134,135,136,137,138,139]. Given the unique characteristics of this outbreak, it is crucial to maintain genomic surveillance efforts to discover and inform genetic changes of the virus in order to develop preventative and control measures in a timely manner.

## Figures and Tables

**Table 1:** Immune evasion genes of the mpox virus.

Protein Function	Gene *
Ankyrin-like protein	OGP037
CC chemokine binding protein	OPG001
TNF and chemokine binding protein, CrmB	OPG002
Ankyrin-like protein	OPG003
Ankyrin-like protein	OPG003
Ankyrin-like protein	OPG015
Ankyrin-like protein	OPG015
OMCP, inhibitor of natural killer cell-mediated NKG2D-dependent cell lysis	OPG016
Viral growth factor; EGF-like protein	OPG019
Apoptosis inhibitor	OPG021
IL-18 binding protein	OPG022
Ankyrin-like protein	OPG023
Ankyrin-like protein	OPG025
Inhibitor of IRF3 and IRF7 activation; BCL-2-like protein	OPG029
Inhibitor of IRF3 and NF-κB activation, apoptosis inhibitor; BCL-2-like protein	OPG035
Ankyrin-like protein	OPG039
Inhibitor of IRF3 NF-κB activation; BCL-2-like protein	OPG044
Apoptosis inhibitor	OPG045
Double-stranded RNA-binding protein, inhibitor of interferon signalling, apoptosis inhibitor	OPG065
Dephosphorylation of STAT1; phosphatase	OPG106
Inhibitor of MHC class II antigen presentation	OPG163
CC and CXC chemokine binding protein	OPG170
Inhibitor of NF-κB activation; BCL-2-like protein	OPG176
Ankyrin-like protein	OPG189
IFNγ binding proteins	OPG193
Inhibitor of intracellular trafficking of MHC class I molecules	OPG195
Apoptosis inhibitor, caspase 1 and caspase 8 inhibitor, SPI-2	OPG199
Inhibitor of NF-κB activation; BCL-2-like protein	OPG200
IFNα/β binding proteins	OPG204
Ankyrin-like protein	OPG205
Apoptosis inhibitor, SPI-1	OPG208

* Genes from a Western African clade mpox virus strain (Accession No. NC_063383). OMCP, orthopoxvirus MHC class I-like protein; EGF, epidermal growth factor; IL, interleukin; IRF, interferon regulatory factor; BCL-2, B cell lymphoma 2; SPI, serine protease inhibitor.

**Table 2:** Frequent amino acid mutations.

Protein	Sub-Virion Location	Site	Mutation	Time of First Appearance
A14L	Membrane	17	A17T	1 September 2018
A19R	Other	435	E435K	1 September 2018
B9R	Secretory	263	L263F	1 September 2018
E6R	Other	606	K606E	1 September 2018
G8L	Other	196	D196N	1 September 2018
H4L	Other	740	H740Y	1 September 2018
L6R	Other	734	S734L	1 September 2018
A19R	Other	62	E62K	1 July 2019
A24R	Other	307	S307L	1 July 2019
J1L	Secretory	105	S105L	1 July 2019
J1R	Other	264	D264N	1 July 2019
J3L	Other	264	D264N	1 July 2019
J3R	Secretory	105	S105L	1 July 2019
A19R	Other	243	R243Q	1 November 2021
B21R	Other	209	D209N	1 November 2021
B21R	Other	1741	M1741I	1 November 2021
C15L	Membrane	78	P78S	1 November 2021
C18L	Membrane	125	E125K	1 November 2021
C9L	Other	48	R48C	1 November 2021
D9L	Secretory	423	A423D	1 November 2021
F8L	Other	108	L108F	1 November 2021
F9R	Membrane	56	D56N	1 November 2021
G9R	Other	30	S30L	1 November 2021
J2L	Secretory	54	S54F	1 November 2021
J2R	Secretory	54	S54F	1 November 2021
A47R	Secretory	221	H221Y	1 January 2022
B21R	Other	722	P722S	1 January 2022
C19L	Membrane	353	E353K	1 January 2022
G10R	Membrane	142	M142I	1 January 2022
G9R	Other	88	D88N	1 January 2022
M4R	Other	162	E162K	1 January 2022

## Data Availability

The datasets have been attached in the supplementary materials and are available from the corresponding author upon reasonable request.

## Supplementary Materials

The following supporting information can be downloaded at: https://www.mdpi.com/article/10.3390/v15040995/s1, Table S1: Frequency of appearance of SNPs.

## References

[1] Cho C.T., Wenner H.A. (1973). Monkeypox virus. Bacteriol. Rev..

[2] Mccollum A.M., Damon I.K. (2013). Human Monkeypox. Clin. Infect. Dis..

[3] Jezek Z., Szczeniowski M., Paluku K.M., Mutombo M. (1987). Human Monkeypox: Clinical Features of 282 Patients. J. Infect. Dis..

[4] CDC Mpox in the U.S.. https://www.cdc.gov/poxvirus/monkeypox/response/2022/world-map.html.

[5] Otu A., Ebenso B., Walley J., Barceló J.M., Ochu C.L. (2022). Global human monkeypox outbreak: Atypical presentation demanding urgent public health action. Lancet Microbe.

[6] Minhaj F.S., Ogale Y.P., Whitehill F., Schultz J., Foote M., Davidson W., Hughes C.M., Wilkins K., Bachmann L., Chatelain R. (2022). Monkeypox Outbreak—Nine States, May 2022. MMWR. Morb. Mortal. Wkly. Rep..

[7] Desingu P.A., Nagarajan K. (2022). Genomic Regions Insertion and Deletion in Monkeypox Virus Causing Multi-Country Out-break-2022. bioRxiv.

[8] Barrett J., McFadden G., Domingo E., Parrish C., Holland J. (2008). Origin and Evolution of Poxviruses.

[9] Hughes A.L., Irausquin S., Friedman R. (2010). The evolutionary biology of poxviruses. Infect. Genet. Evol..

[10] Schmidt F.I., Bleck C.K.E., Mercer J. (2012). Poxvirus host cell entry. Curr. Opin. Virol..

[11] Kilcher S., Schmidt F.I., Schneider C., Kopf M., Helenius A., Mercer J. (2014). siRNA Screen of Early Poxvirus Genes Identifies the AAA+ ATPase D5 as the Virus Genome-Uncoating Factor. Cell Host Microbe.

[12] Moss B. (2006). Poxvirus entry and membrane fusion. Virology.

[13] Moss B. (1991). Vaccinia Virus: A Tool for Research and Vaccine Development. Science.

[14] Payne L.G. (1980). Significance of Extracellular Enveloped Virus in the in vitro and in vivo Dissemination of Vaccinia. J. Gen. Virol..

[15] Shchelkunov S.N., Totmenin A.V., Babkin I.V., Safronov P.F., Ryazankina O.I., Petrov N.A., Gutorov V.V., Uvarova E., Mikheev M.V., Sisler J.R. (2001). Human monkeypox and smallpox viruses: Genomic comparison. FEBS Lett..

[16] Kugelman J.R., Johnston S.C., Mulembakani P.M., Kisalu N., Lee M.S., Koroleva G., McCarthy S.E., Gestole M.C., Wolfe N.D., Fair J.N. (2014). Genomic Variability of Monkeypox Virus among Humans, Democratic Republic of the Congo. Emerg. Infect. Dis..

[17] Seet B.T., Johnston J.B., Brunetti C.R., Barrett J.W., Everett H., Cameron C., Sypula J., Nazarian S.H., Lucas A., McFadden G. (2003). Poxviruses and Immune Evasion. Ann. Rev. Immunol..

[18] Reed K.D., Melski J.W., Graham M.B., Regnery R.L., Sotir M.J., Wegner M.V., Kazmierczak J.J., Stratman E.J., Li Y., Fairley J.A. (2004). The Detection of Monkeypox in Humans in the Western Hemisphere. N. Engl. J. Med..

[19] Resch W., Hixson K.K., Moore R.J., Lipton M.S., Moss B. (2006). Protein composition of the vaccinia virus mature virion. Virology.

[20] Manes N.P., Estep R.D., Mottaz H.M., Moore R.J., Clauss T.R.W., Monroe M.E., Du X., Adkins J.N., Wong S.W., Smith R.D. (2008). Comparative Proteomics of Human Monkeypox and Vaccinia Intracellular Mature and Extracellular Enveloped Virions. J. Proteome Res..

[21] Shchelkunov S., Totmenin A., Safronov P., Mikheev M., Gutorov V., Ryazankina O., Petrov N., Babkin I., Uvarova E., Sandakhchiev L. (2002). Analysis of the Monkeypox Virus Genome. Virology.

[22] Chen N., Li G., Liszewski M.K., Atkinson J.P., Jahrling P.B., Feng Z., Schriewer J., Buck C., Wang C., Lefkowitz E.J. (2005). Virulence differences between monkeypox virus isolates from West Africa and the Congo basin. Virology.

[23] Weaver J.R., Isaacs S.N. (2008). Monkeypox virus and insights into its immunomodulatory proteins. Immunol. Rev..

[24] Reynolds M.G., Damon I.K. (2012). Outbreaks of human monkeypox after cessation of smallpox vaccination. Trends Microbiol..

[25] McFadden G. (2005). Poxvirus tropism. Nat. Rev. Genet..

[26] Smith G.L., Law M. (2004). The exit of Vaccinia virus from infected cells. Virus Res..

[27] Pickup D.J. (2015). Extracellular Virions: The Advance Guard of Poxvirus Infections. PLoS Pathog..

[28] Hsiao J.-C., Chung C.-S., Chang W. (1999). Vaccinia Virus Envelope D8L Protein Binds to Cell Surface Chondroitin Sulfate and Mediates the Adsorption of Intracellular Mature Virions to Cells. J. Virol..

[29] Chung C.-S., Hsiao J.-C., Chang Y.-S., Chang W. (1998). A27L Protein Mediates Vaccinia Virus Interaction with Cell Surface Heparan Sulfate. J. Virol..

[30] Lin C.-L., Chung C.-S., Heine H., Chang W. (2000). Vaccinia Virus Envelope H3L Protein Binds to Cell Surface Heparan Sulfate and Is Important for Intracellular Mature Virion Morphogenesis and Virus Infection In Vitro and In Vivo. J. Virol..

[31] Moss B. (2016). Membrane fusion during poxvirus entry. Semin. Cell Dev. Biol..

[32] Khodakevich L., Jezek Z., Messinger D. (1988). Monkeypox virus—Ecology and public-health significance. Bull. World Health Organ..

[33] Reynolds M.G., Suu-Ire R., Karem K., Root J.J., Galley J., Carroll D.S., Abel J., Kwasi M.O., Damon I.K., Likos A. (2010). A Silent Enzootic of an Orthopoxvirus in Ghana, West Africa: Evidence for Multi-Species Involvement in the Absence of Widespread Human Disease. Am. J. Trop. Med. Hyg..

[34] Salzer J.S., Carroll D.S., Rwego I.B., Li Y., Falendysz E.A., Shisler J.L., Karem K.L., Damon I.K., Gillespie T.R. (2013). Serologic Evidence For Circulating Orthopoxviruses In Peridomestic Rodents From Rural Uganda. J. Wildl. Dis..

[35] Orba Y., Sasaki M., Yamaguchi H., Ishii A., Thomas Y., Ogawa H., Hang’ombe B.M., Mweene A.S., Morikawa S., Saijo M. (2015). Orthopoxvirus infection among wildlife in Zambia. J. Gen. Virol..

[36] Doty J.B., Malekani J.M., Kalemba L.N., Stanley W.T., Monroe B.P., Nakazawa Y.U., Mauldin M.R., Bakambana T.L., Liyandja T.L.D., Braden Z.H. (2017). Assessing Monkeypox Virus Prevalence in Small Mammals at the Human–Animal Interface in the Democratic Republic of the Congo. Viruses.

[37] Hutin Y.J., Williams R.J., Malfait P., Pebody R., Loparev V.N., Ropp S.L., Rodriguez M., Knight J.C., Tshioko F.K., Khan A.S. (2001). Outbreak of Human Monkeypox, Democratic Republic of Congo, 1996 to 1997. Emerg. Infect. Dis..

[38] Hutson C.L., Olson V.A., Carroll D.S., Abel J.A., Hughes C.M., Braden Z.H., Weiss S., Self J., Osorio J.E., Hudson P.N. (2009). A prairie dog animal model of systemic orthopoxvirus disease using West African and Congo Basin strains of monkeypox virus. J. Gen. Virol..

[39] Yinka-Ogunleye A., Aruna O., Dalhat M., Ogoina D., McCollum A., Disu Y., Mamadu I., Akinpelu A., Ahmad A., Burga J. (2019). Outbreak of human monkeypox in Nigeria in 2017–18: A clinical and epidemiological report. Lancet Infect. Dis..

[40] Falendysz E.A., Lopera J.G., Doty J.B., Nakazawa Y., Crill C., Lorenzsonn F., Kalemba L.N., Ronderos M.D., Mejia A., Malekani J.M. (2017). Characterization of Monkeypox virus infection in African rope squirrels (Funisciurus sp.). PLoS Neglected Trop. Dis..

[41] Keasey S., Pugh C., Tikhonov A., Chen G., Schweitzer B., Nalca A., Ulrich R.G. (2010). Proteomic Basis of the Antibody Response to Monkeypox Virus Infection Examined in Cynomolgus Macaques and a Comparison to Human Smallpox Vaccination. PLoS ONE.

[42] Reynolds M.G., Doty J.B., Mccollum A.M., Olson V.A., Nakazawa Y. (2018). Monkeypox re-emergence in Africa: A call to expand the concept and practice of One Health. Expert Rev. Anti-infective Ther..

[43] Zaucha G.M., Jahrling P.B., Geisbert T.W., Swearengen J.R., Hensley L. (2001). The Pathology of Experimental Aerosolized Monkeypox Virus Infection in Cynomolgus Monkeys (*Macaca fascicularis*). Lab. Investig..

[44] Hammarlund E., Dasgupta A., Pinilla C., Norori P., Früh K., Slifka M.K. (2008). Monkeypox virus evades antiviral CD4 ^+^ and CD8 ^+^ T cell responses by suppressing cognate T cell activation. Proc. Natl. Acad. Sci. USA.

[45] Rubins K.H., Hensley L.E., Jahrling P.B., Whitney A.R., Geisbert T.W., Huggins J.W., Owen A., LeDuc J.W., Brown P.O., Relman D.A. (2004). The host response to smallpox: Analysis of the gene expression program in peripheral blood cells in a nonhuman primate model. Proc. Natl. Acad. Sci. USA.

[46] Jahrling P.B., Hensley L.E., Martinez M.J., LeDuc J.W., Rubins K.H., Relman D.A., Huggins J.W. (2004). Exploring the potential of variola virus infection of cynomolgus macaques as a model for human smallpox. Proc. Natl. Acad. Sci. USA.

[47] Rubins K.H., Hensley L.E., Relman D.A., Brown P.O. (2011). Stunned Silence: Gene Expression Programs in Human Cells Infected with Monkeypox or Vaccinia Virus. PLoS ONE.

[48] Paust S., Senman B., Von Andrian U.H. (2010). Adaptive immune responses mediated by natural killer cells. Immunol. Rev..

[49] Song H., Josleyn N., Janosko K., Skinner J., Reeves R.K., Cohen M., Jett C., Johnson R., Blaney J.E., Bollinger L. (2013). Monkeypox Virus Infection of Rhesus Macaques Induces Massive Expansion of Natural Killer Cells but Suppresses Natural Killer Cell Functions. PLoS ONE.

[50] Johnston S.C., Lin K.L., Connor J.H., Ruthel G., Goff A., Hensley L.E. (2012). In vitro inhibition of monkeypox virus production and spread by Interferon-β. Virol. J..

[51] Johnston S.C., Johnson J.C., Stonier S.W., Lin K.L., Kisalu N.K., Hensley L.E., Rimoin A.W. (2015). Cytokine modulation correlates with severity of monkeypox disease in humans. J. Clin. Virol..

[52] Liu L., Xu Z., Fuhlbrigge R.C., Peña-Cruz V., Lieberman J., Kupper T.S. (2005). Vaccinia Virus Induces Strong Immunoregulatory Cytokine Production in Healthy Human Epidermal Keratinocytes: A Novel Strategy for Immune Evasion. J. Virol..

[53] Howell M.D., Gallo R.L., Boguniewicz M., Jones J.F., Wong C., Streib J.E., Leung D.Y. (2006). Cytokine Milieu of Atopic Dermatitis Skin Subverts the Innate Immune Response to Vaccinia Virus. Immunity.

[54] Broek M.V.D., Bachmann M.F., Köhler G., Barner M., Escher R., Zinkernagel R., Kopf M. (2000). IL-4 and IL-10 Antagonize IL-12-Mediated Protection Against Acute Vaccinia Virus Infection with a Limited Role of IFN-γ and Nitric Oxide Synthetase 2. J. Immunol..

[55] Thakur A., Mikkelsen H., Jungersen G. (2019). Intracellular Pathogens: Host Immunity and Microbial Persistence Strategies. J. Immunol. Res..

[56] Strassburg M.A. (1982). The global eradication of smallpox. Am. J. Infect. Control..

[57] Cherry J.D., McIntosh K., Connor J.D., Benenson A.S., Alling D.W., Rolfe U.T., Todd W.A., Schanberger J.E., Mattheis M.J. (1977). Primary Percutaneous Vaccination. J. Infect. Dis..

[58] Edghill-Smith Y., Golding H., Manischewitz J., King L.R., Scott D., Bray M., Nalca A., Hooper J., Whitehouse C.A., Schmitz J.E. (2005). Smallpox vaccine–induced antibodies are necessary and sufficient for protection against monkeypox virus. Nat. Med..

[59] Jacobs B.L., Langland J.O., Kibler K.V., Denzler K.L., White S.D., Holechek S.A., Wong S., Huynh T., Baskin C.R. (2009). Vaccinia virus vaccines: Past, present and future. Antivir. Res..

[60] Crotty S., Felgner P., Davies H., Glidewell J., Villarreal L., Ahmed R. (2003). Cutting Edge: Long-Term B Cell Memory in Humans after Smallpox Vaccination. J. Immunol..

[61] Hammarlund E., Lewis M.W., Hansen S.G., Strelow L.I., Nelson J.A., Sexton G.J., Hanifin J.M., Slifka M.K. (2003). Duration of antiviral immunity after smallpox vaccination. Nat. Med..

[62] Mack T.M., Noble J., Thomas D.B. (1972). A Prospective Study of Serum Antibody and Protection Against Smallpox. Am. J. Trop. Med. Hyg..

[63] MacLeod M.K., Clambey E.T., Kappler J.W., Marrack P. (2009). CD4 memory T cells: What are they and what can they do?. Semin. Immunol..

[64] Hickman H.D., Reynoso G.V., Ngudiankama B.F., Rubin E.J., Magadán J.G., Cush S.S., Gibbs J., Molon B., Bronte V., Bennink J.R. (2013). Anatomically Restricted Synergistic Antiviral Activities of Innate and Adaptive Immune Cells in the Skin. Cell Host Microbe.

[65] Marco M.D.M.F.D., Alejo A., Hudson P., Damon I.K., Alcami A. (2009). The highly virulent variola and monkeypox viruses express secreted inhibitors of type I interferon. FASEB J..

[66] Esteban D.J., Nuara A.A., Buller R.M.L. (2004). Interleukin-18 and glycosaminoglycan binding by a protein encoded by Variola virus. J. Gen. Virol..

[67] Takata M.A., Gonçalves-Carneiro D., Zang T.M., Soll S.J., York A., Blanco-Melo D., Bieniasz P.D. (2017). CG dinucleotide suppression enables antiviral defence targeting non-self RNA. Nature.

[68] Kmiec D., Nchioua R., Sherrill-Mix S., Stürzel C.M., Heusinger E., Braun E., Gondim M.V.P., Hotter D., Sparrer K.M.J., Hahn B.H. (2020). CpG Frequency in the 5′ Third of the *env* Gene Determines Sensitivity of Primary HIV-1 Strains to the Zinc-Finger Antiviral Protein. Mbio.

[69] Nchioua R., Kmiec D., Müller J.A., Conzelmann C., Groß R., Swanson C.M., Neil S.J.D., Stenger S., Sauter D., Münch J. (2020). SARS-CoV-2 Is Restricted by Zinc Finger Antiviral Protein despite Preadaptation to the Low-CpG Environment in Humans. mBio.

[70] Peng C., Wyatt L.S., Glushakow-Smith S.G., Lal-Nag M., Weisberg A.S., Moss B. (2020). Zinc-finger antiviral protein (ZAP) is a restriction factor for replication of modified vaccinia virus Ankara (MVA) in human cells. PLoS Pathog..

[71] Hudson P.N., Self J., Weiss S., Braden Z., Xiao Y., Girgis N.M., Emerson G., Hughes C., Sammons S.A., Isaacs S.N. (2012). Elucidating the Role of the Complement Control Protein in Monkeypox Pathogenicity. PLoS ONE.

[72] Bunge E.M., Hoet B., Chen L., Lienert F., Weidenthaler H., Baer L.R., Steffen R. (2022). The changing epidemiology of human monkeypox—A potential threat? A systematic review. PLoS Neglected Trop. Dis..

[73] Nolen L.D., Tamfum J.-J.M., Kabamba J., Likofata J., Katomba J., McCollum A.M., Monroe B., Kalemba L., Mukadi D., Bomponda P.L. (2015). Introduction of Monkeypox into a Community and Household: Risk Factors and Zoonotic Reservoirs in the Democratic Republic of the Congo. Am. J. Trop. Med. Hyg..

[74] Reynolds M.G., Davidson W.B., Curns A.T., Conover C.S., Huhn G., Davis J.P., Wegner M., Croft D.R., Newman A., Obiesie N.N. (2007). Spectrum of Infection and Risk Factors for Human Monkeypox, United States, 2003. Emerg. Infect. Dis..

[75] HAN Archive—00446 | Health Alert Network (HAN). https://emergency.cdc.gov/han/2021/han00446.asp.

[76] Sklenovská N., Van Ranst M. (2018). Emergence of Monkeypox as the Most Important Orthopoxvirus Infection in Humans. Front. Public Heal..

[77] Von Magnus P., Andersen E.K., Petersen K.B., Birch-Andersen A. (2009). A pox-like disease in cynomolgus monkeys. Acta Pathol. Microbiol. Scand..

[78] Marennikova S.S., Seluhina E.M., Mal’Ceva N.N., Cimiskjan K.L., Macevic G.R. (1972). Isolation and properties of the causal agent of a new variola-like disease (monkeypox) in man. Bull. World Health Organ..

[79] Ladnyj I., Ziegler P., Kima E. (1972). Human infection caused by monkeypox virus in basankusu terri-tory, democratic-republic-of-congo. Bull. World Health Organ..

[80] Foster S., Eke R., Foege W., Smith E., Titus J., Moser C., Lourie B., Brink E., Kuteyi O., Cummings E. (1972). HUMAN MONKEYPOX. Bull. World Health Organ..

[81] Huhn G.D., Bauer A.M., Yorita K., Graham M.B., Sejvar J., Likos A., Damon I.K., Reynolds M., Kuehnert M.J. (2005). Clinical Characteristics of Human Monkeypox, and Risk Factors for Severe Disease. Clin. Infect. Dis..

[82] Fleischauer A.T., Kile J.C., Davidson M., Fischer M., Karem K.L., Teclaw R., Messersmith H., Pontones P., Beard B.A., Braden Z.H. (2005). Evaluation of Human-to-Human Transmission of Monkeypox from Infected Patients to Health Care Workers. Clin. Infect. Dis..

[83] Adler H., Gould S., Hine P., Snell L.B., Wong W., Houlihan C.F., Osborne J.C., Rampling T., Beadsworth M.B., Duncan C.J. (2022). Clinical features and management of human monkeypox: A retrospective observational study in the UK. Lancet Infect. Dis..

[84] Bragazzi N.L., Kong J.D., Mahroum N., Tsigalou C., Khamisy-Farah R., Converti M., Wu J. (2022). Epidemiological trends and clinical features of the ongoing monkeypox epidemic: A preliminary pooled data analysis and literature review. J. Med. Virol..

[85] Multi-Country Monkeypox Outbreak: Situation Update. https://www.who.int/emergencies/disease-outbreak-news/item/2022-DON390.

[86] Nguyen P.-Y., Ajisegiri W.S., Costantino V., Chughtai A.A., MacIntyre C.R. (2021). Reemergence of Human Monkeypox and Declining Population Immunity in the Context of Urbanization, Nigeria, 2017–2020. Emerg. Infect. Dis..

[87] Petersen E., Kantele A., Koopmans M., Asogun D., Yinka-Ogunleye A., Ihekweazu C., Zumla A. (2019). Human monkeypox: Epidemiologic and clinical characteristics, diagnosis, and prevention. Infect. Dis. Clin..

[88] Simpson K., Heymann D., Brown C.S., Edmunds W.J., Elsgaard J., Fine P., Hochrein H., Hoff N.A., Green A., Ihekweazu C. (2020). Human monkeypox—After 40 years, an unintended consequence of smallpox eradication. Vaccine.

[89] Reynolds M.G., Carroll D.S., Karem K.L. (2012). Factors affecting the likelihood of monkeypox’s emergence and spread in the post-smallpox era. Curr. Opin. Virol..

[90] Likos A.M., Sammons S.A., Olson V.A., Frace A.M., Li Y., Olsen-Rasmussen M., Davidson W., Galloway R., Khristova M.L., Reynolds M.G. (2005). A tale of two clades: Monkeypox viruses. J. Gen. Virol..

[91] Beer E.M., Rao V.B. (2019). A systematic review of the epidemiology of human monkeypox outbreaks and implications for outbreak strategy. PLoS Neglected Trop. Dis..

[92] Hadfield J., Megill C., Bell S.M., Huddleston J., Potter B., Callender C., Sagulenko P., Bedford T., Neher R.A. (2018). Nextstrain: Real-time tracking of pathogen evolution. Bioinformatics.

[93] Capella-Gutiérrez S., Silla-Martínez J.M., Gabaldón T. (2009). trimAl: A tool for automated alignment trimming in large-scale phylogenetic analyses. Bioinformatics.

[94] Nguyen L.-T., Schmidt H.A., Von Haeseler A., Minh B.Q. (2015). IQ-TREE: A Fast and Effective Stochastic Algorithm for Estimating Maximum-Likelihood Phylogenies. Mol. Biol. Evol..

[95] Kalyaanamoorthy S., Minh B.Q., Wong T.K.F., Von Haeseler A., Jermiin L.S. (2017). ModelFinder: Fast model selection for accurate phylogenetic estimates. Nat. Methods.

[96] Hoang D.T., Chernomor O., Von Haeseler A., Minh B.Q., Vinh L.S. (2018). UFBoot2: Improving the Ultrafast Bootstrap Approximation. Mol. Biol. Evol..

[97] Letunic I., Bork P. (2006). Interactive Tree Of Life (iTOL): An online tool for phylogenetic tree display and annotation. Bioinformatics.

[98] Letunic I., Bork P. (2021). Interactive Tree of Life (iTOL) v5: An online tool for phylogenetic tree display and annotation. Nucleic Acids Res..

[99] Vaughan A., Aarons E., Astbury J., Balasegaram S., Beadsworth M., Beck C.R., Chand M., O’connor C., Dunning J., Ghebrehewet S. (2018). Two cases of monkeypox imported to the United Kingdom, September 2018. Eurosurveillance.

[100] Erez N., Achdout H., Milrot E., Schwartz Y., Wiener-Well Y., Paran N., Politi B., Tamir H., Israely T., Weiss S. (2019). Diagnosis of Imported Monkeypox, Israel, 2018. Emerg. Infect. Dis..

[101] Yong S.E.F., Ng O.T., Ho Z.J.M., Mak T.M., Marimuthu K., Vasoo S., Yeo T.W., Ng Y.K., Cui L., Ferdous Z. (2020). Imported Monkeypox, Singapore. Emerg. Infect. Dis..

[102] Vaughan A., Aarons E., Astbury J., Brooks T., Chand M., Flegg P., Hardman A., Harper N., Jarvis R., Mawdsley S. (2020). Human-to-Human Transmission of Monkeypox Virus, United Kingdom, October 2018. Emerg. Infect. Dis..

[103] Moss B. (2013). Poxvirus DNA Replication. Cold Spring Harb. Perspect. Biol..

[104] Firth C., Kitchen A., Shapiro B., Suchard M.A., Holmes E.C., Rambaut A. (2010). Using Time-Structured Data to Estimate Evolutionary Rates of Double-Stranded DNA Viruses. Mol. Biol. Evol..

[105] Duggan A.T., Perdomo M.F., Piombino-Mascali D., Marciniak S., Poinar D., Emery M.V., Buchmann J.P., Duchêne S., Jankauskas R., Humphreys M. (2016). 17th Century Variola Virus Reveals the Recent History of Smallpox. Curr. Biol..

[106] Duffy S., Shackelton L.A., Holmes E.C. (2008). Rates of evolutionary change in viruses: Patterns and determinants. Nat. Rev. Genet..

[107] Isidro J., Borges V., Pinto M., Sobral D., Santos J.D., Nunes A., Mixão V., Ferreira R., Santos D., Duarte S. (2022). Phylogenomic characterization and signs of microevolution in the 2022 multi-country outbreak of monkeypox virus. Nat. Med..

[108] Jern P., Russell R.A., Pathak V., Coffin J.M. (2009). Likely Role of APOBEC3G-Mediated G-to-A Mutations in HIV-1 Evolution and Drug Resistance. PLoS Pathog..

[109] Martinez T., Shapiro M., Bhaduri-McIntosh S., MacCarthy T. (2019). Evolutionary effects of the AID/APOBEC family of mutagenic enzymes on human gamma-herpesviruses. Virus Evol..

[110] Kremer M., Suezer Y., Martinez-Fernandez Y., Münk C., Sutter G., Schnierle B.S. (2006). Vaccinia virus replication is not affected by APOBEC3 family members. Virol. J..

[111] Giorgi F.M., Pozzobon D., Meglio A.D., Mercatelli D. (2022). Genomic Analysis of the Recent Monkeypox Outbreak. Med.

[112] Gubser C., Hué S., Kellam P., Smith G.L. (2004). Poxvirus genomes: A phylogenetic analysis. J. Gen. Virol..

[113] Pérez-Losada M., Arenas M., Galán J.C., Palero F., González-Candelas F. (2015). Recombination in viruses: Mechanisms, methods of study, and evolutionary consequences. Infect. Genet. Evol..

[114] Esposito J.J., Sammons S.A., Frace A.M., Osborne J.D., Olsen-Rasmussen M., Zhang M., Govil D., Damon I.K., Kline R., Laker M. (2006). Genome Sequence Diversity and Clues to the Evolution of Variola (Smallpox) Virus. Science.

[115] Coulson D., Upton C. (2010). Characterization of indels in poxvirus genomes. Virus Genes.

[116] Evans D.H. (2022). Poxvirus Recombination. Pathogens.

[117] Sasani T.A., Cone K.R., Quinlan A.R., Elde N.C. (2018). Long read sequencing reveals poxvirus evolution through rapid homogenization of gene arrays. Elife.

[118] Cone K.R., Kronenberg Z.N., Yandell M., Elde N.C. (2017). Emergence of a Viral RNA Polymerase Variant during Gene Copy Number Amplification Promotes Rapid Evolution of Vaccinia Virus. J. Virol..

[119] Yeh T.-Y., Hsieh Z.-Y., Feehley M.C., Feehley P.J., Contreras G.P., Su Y.-C., Hsieh S.-L., Lewis D.A. (2022). Recombination shapes the 2022 monkeypox (mpox) outbreak. Med.

[120] Estep R.D., Messaoudi I., O’Connor M.A., Li H., Sprague J., Barron A., Engelmann F., Yen B., Powers M.F., Jones J.M. (2011). Deletion of the Monkeypox Virus Inhibitor of Complement Enzymes Locus Impacts the Adaptive Immune Response to Monkeypox Virus in a Nonhuman Primate Model of Infection. J. Virol..

[121] Goff A., Mucker E., Raymond J., Fisher R., Bray M., Hensley L., Paragas J. (2011). Infection of cynomolgus macaques with a recombinant monkeypox virus encoding green fluorescent protein. Arch. Virol..

[122] Hughes A.L., Friedman R. (2005). Poxvirus genome evolution by gene gain and loss. Mol. Phylogenet. Evol..

[123] Elde N.C., Child S.J., Eickbush M.T., Kitzman J.O., Rogers K.S., Shendure J., Geballe A.P., Malik H.S. (2012). Poxviruses Deploy Genomic Accordions to Adapt Rapidly against Host Antiviral Defenses. Cell.

[124] Hendrickson R.C., Wang C., Hatcher E.L., Lefkowitz E.J. (2010). Orthopoxvirus Genome Evolution: The Role of Gene Loss. Viruses.

[125] Meyer H., Sutter G., Mayr A. (1991). Mapping of deletions in the genome of the highly attenuated vaccinia virus MVA and their influence on virulence. J. Gen. Virol..

[126] Hatch G.J., Graham V.A., Bewley K.R., Tree J.A., Dennis M., Taylor I., Funnell S.G.P., Bate S.R., Steeds K., Tipton T. (2013). Assessment of the Protective Effect of Imvamune and Acam2000 Vaccines against Aerosolized Monkeypox Virus in Cynomolgus Macaques. J. Virol..

[127] Parker S., Buller R.M. (2013). A review of experimental and natural infections of animals with monkeypox virus between 1958 and 2012. Future Virol..

[128] Hatcher E.L., Hendrickson R.C., Lefkowitz E.J. (2014). Identification of Nucleotide-Level Changes Impacting Gene Content and Genome Evolution in Orthopoxviruses. J. Virol..

[129] Brinkmann A., Kohl C., Pape K., Bourquain D., Thürmer A., Michel J., Schaade L., Nitsche A. (2022). Possible Adaption of the 2022 Monkeypox Virus to the Human Host through Gene Duplication and Loss. bioRxiv.

[130] Yang Z. (2022). Monkeypox: A potential global threat?. J. Med. Virol..

[131] Fine P.E.M., Jezek Z., Grab B., Dixon H. (1988). The Transmission Potential of Monkeypox Virus in Human Populations. Leuk. Res..

[132] Fenner F. (1980). The Global Eradication Of Smallpox. Med. J. Aust..

[133] Karim S.S.A., Karim Q.A. (2021). Omicron SARS-CoV-2 variant: A new chapter in the COVID-19 pandemic. Lancet..

[134] Sharma A., Ahmad Farouk I., Lal S.K. (2021). COVID-19: A Review on the Novel Coronavirus Disease Evolution, Transmission, Detection, Control and Prevention. Viruses.

[135] Liu Y., Liu J., Du S., Shan C., Nie K., Zhang R., Li X.-F., Zhang R., Wang T., Qin C.-F. (2017). Evolutionary enhancement of Zika virus infectivity in Aedes aegypti mosquitoes. Nature.

[136] Yu X., Shan C., Zhu Y., Ma E., Wang J., Wang P., Shi P.-Y., Cheng G. (2021). A mutation-mediated evolutionary adaptation of Zika virus in mosquito and mammalian host. Proc. Natl. Acad. Sci. USA.

[137] Yu X., Cheng G. (2022). Contribution of phylogenetics to understanding the evolution and epidemiology of dengue virus. Anim. Model. Exp. Med..

[138] Yu X., Cheng G. (2022). Adaptive Evolution as a Driving Force of the Emergence and Re-Emergence of Mosquito-Borne Viral Diseases. Viruses.

[139] Chen L., Zhang X., Guo X., Peng W., Zhu Y., Wang Z., Yu X., Shi H., Li Y., Zhang L. (2022). Neighboring mutation-mediated enhancement of dengue virus infectivity and spread. EMBO Rep..

